# Flood hazard management in a multiple hazard context: a systematic review of flood hazard management during the COVID-19 pandemic in Africa

**DOI:** 10.1007/s43832-022-00014-w

**Published:** 2022-04-19

**Authors:** Bashiru Turay

**Affiliations:** 1 Department of Geography, University of Bonn, Bonn, Germany; 2 Institute for Environment and Human Security, United Nations University (UNU-EHS), Bonn, Germany

**Keywords:** Flood management, Multiple hazards, Africa, COVID-19 pandemic, Disaster risk, Impacts

## Abstract

Result-oriented research can uncover hidden flood management obstacles and propose solutions that, if combined with political will, appropriate technology, and resources, can overcome the majority of Africa’s future flood calamities. In view of this, it is critical to examine researchers' findings on flood hazard management, particularly now that the continent is struggling with COVID-19 and other hazards. This study employed a systematic review approach to critically analyze 103 contextually detailed studies with a set of criteria that were not only meant to keep the focus on floods and the COVID-19 pandemic but also to understand the context of managing floods during COVID-19 and other hazards at the same time on the continent. I found that the authors strongly recommend how institutions should create non-structural enabling environments for managing combined hazards. Also, researchers paid little attention to recommending ecosystem-based measures for flood management during the COVID-19 pandemic in Africa. Future research should study how different countries in Africa are preparing to manage multiple future hazards, including the comparative assessment of the strengths and weaknesses of individual countries’ planning and preparation.

## Introduction

Africa comprises fifty-four countries with a wide range of climatic extremes, from equatorial to desert [22]. Flooding is the most common natural hazard on the continent, and it frequently causes property damage and human deaths [9].

The continent has the most vulnerable flood populations, as they lack the resources and understanding developed countries have. The majority of these vulnerable people live in Sub-Saharan Africa, where 55 per cent of the population lives in extreme poverty and is at serious risk of flooding [33].

The Coronavirus disease was declared a global disaster on 11 March 2020 but had already reached Africa with its devastating effects in February of the same year. The COVID-19 pandemic adds to the fragility of Africa's already frail and unstable, health systems. Maintaining vital services is complicated with a limited number of medical workers, equipment, supplies, and infrastructure [21].

As the pandemic spreads, structural vulnerabilities such as poor productivity, high unemployment, and high inequality spread, with deterioration of the limited fiscal capacity and no signs of improvement even as some African countries began to recover [23].

Africa's countries plagued by conflict and violence are the most vulnerable to the pandemic's effects. In the Sahel alone, over 1,000 violent episodes occurred in 2019, claiming 8,000 lives and displacing 1.5 million people. As many as 18 million people have been displaced by conflicts and violence in the afflicted nations [23]. Countries in the Horn of Africa have suffered from the severe social, economic, and environmental consequences of a long-term refugee presence. Aside from the deadly pandemic, locust swarms, regional insecurity, and warfare, climate-change-related droughts and flooding have damaged crops and livelihoods of millions of smallholder farmers in the Horn, worsening the existing food security problems [20, 23]. In fragile and conflict-affected areas, the pandemic will push an extra estimated 18 to 29 million people into extreme poverty [23].

The COVID-19 outbreak's crippling effects are predicted to last the longest in countries that are least capable to build resilience [3]. Even though the impact of the disease is vividly seen, it is framed as fake or as part of selfish political agendas. The pandemic's politicization, combined with contradictory falsehoods, hindered the motivation to act and the ability and mandate [42].

This paper adopted the definition of multiple hazards by the United Nations, which refers to them as situations in which hazardous occurrences may occur concurrently, cascadingly, or cumulatively over time, considering the potential associated impacts [43].

Flood hazard management, as used in this work, refers to flood interventions in Africa. These include various activities, projects, and programs ranging from food aid to victims to the development of drainage systems at differing stages of a flood event, from hazard mitigation and preparedness to acute humanitarian relief, community recovery, and long-term climate adaptation.

Managing floods amid the COVID-19 epidemic is increasingly difficult. When responders are addressing many disasters simultaneously, the activities to control one can exaggerate the other [3]. Improper flood response during the pandemic might hasten the spread of COVID-19 and intensify its consequences, leading to more human deaths and socioeconomic damage [3]. Flood emergency response, such as evacuations, might be complicated by COVID-19 measures like social distancing and isolation [45]. Under tight COVID-19 rules, flood hazard response may be insufficient to reduce damage [3].

Research emphasizing the relationship between flood hazards and the impacts on the African continent is long-standing [5, 17, 26, 32, 33, 37]. Wagner and others [44] did a systematic review of flood management in West Africa. According to their findings, flood management research in the region is shifting from flood protection to flood risk management. They further pointed out the necessity of addressing flood risk through an integrated strategy containing structural and non-structural measures and considering residual risks [44]. The role of the COVID-19 pandemic on the continent is also well researched [21, 23].

The combination of different hazards at the same time and space in different parts of the continent has turned the attention of researchers from investigating single hazards to investigating multiple hazards and disasters. For example, Kassegn and Endris investigated the socioeconomic impacts of COVID-19, desert locusts, and floods in East Africa, concluding that the triple threats exacerbated existing food insecurity and undermined livelihoods and development gains that have taken years to build [16]. Tiepolo and his colleagues conducted a multi-hazard risk assessment in Niger. They discovered that the geographical distribution of the country's adaptation and resilience programs does not reflect the risk level, and that one-third of the local development plans reviewed advocate activities that are incompatible with the major hydroclimatic dangers [41].

Despite all the advances made so far; limited knowledge exists for understanding flood hazards management amid the COVID-19 pandemic on the African continent. I wish to contribute to filling this gap by critically reviewing studies relevant to this subject.

The majority of the future flood control obstacles that the African continent will encounter may be addressed by effective research backed by political will, appropriate technology, and resources [22]. In light of this, it is essential to examine researchers' findings on flood hazard management, particularly now when the continent is battling with COVID-19 and other hazards.

An analysis of the relevant hazards, authors’ recommendations for flood management, and how flood hazards were managed in multiple hazard circumstances during the COVID-19 pandemic on the continent were adequately presented in the respective sections of this paper.

## Methodology

This study employed a systematic methodology to critically review the relevant knowledge using the following set of criteria:

### Inclusion criteria

For inclusion, the paper must address flooding, COVID-19, and other hazards and disastrous events compounded with flooding and COVID-19. The article must include research about Africa or have part of the continent in its assessment. Only papers published in English between 2010 and 2021 were considered. The stated criteria are not only to keep the focus on floods and the COVID-19 pandemic but also to understand the context of managing floods during COVID-19 and other hazards at the same time on the continent.

### Search strategy

I did an advanced search in the Web of science with the following search strings: (((((TS = (flooding and Covid-19 pandemic in Africa)) OR TS = (floods in Africa)) OR TS = (inundation, flood risk, and hazard management in Africa)) OR TS = (flood response, coping, adaptation and resilience in Africa)) OR TS = (multiple hazards, multi-hazards, compounding hazards and flood disaster management in Africa)) OR TS = (COVID-19, coronavirus disease management, SARS-CoV-2 virus disease in African countries). The search string contained a combination of keyword synonyms to capture a broad but manageable coverage of papers relevant to the research topic.

### Screening

**Fig. 1:**
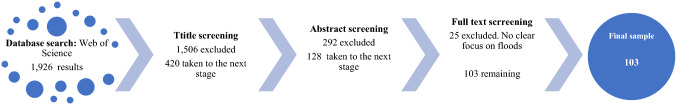
Review methodology

Title screening was done to ensure the selected papers met the inclusion criteria mentioned above. After the title screening, 420 articles remained. The abstract sections of the remaining articles were further screened. After the abstract screening process, a total of 128 papers remained. At this stage, I read the articles in their entirety. The complete reading of the documents led to the omission of 25 that did not satisfy the study’s criteria for inclusion. A final sample of 103 articles was selected for this research. The chosen selection is contextually explicit and consistent with the study’s objective. Figure 1 is an illustration of the literature review methodology.

## Results and discussion

### Characteristics of studies reviewed

#### Spatial distribution

**Fig. 2:**
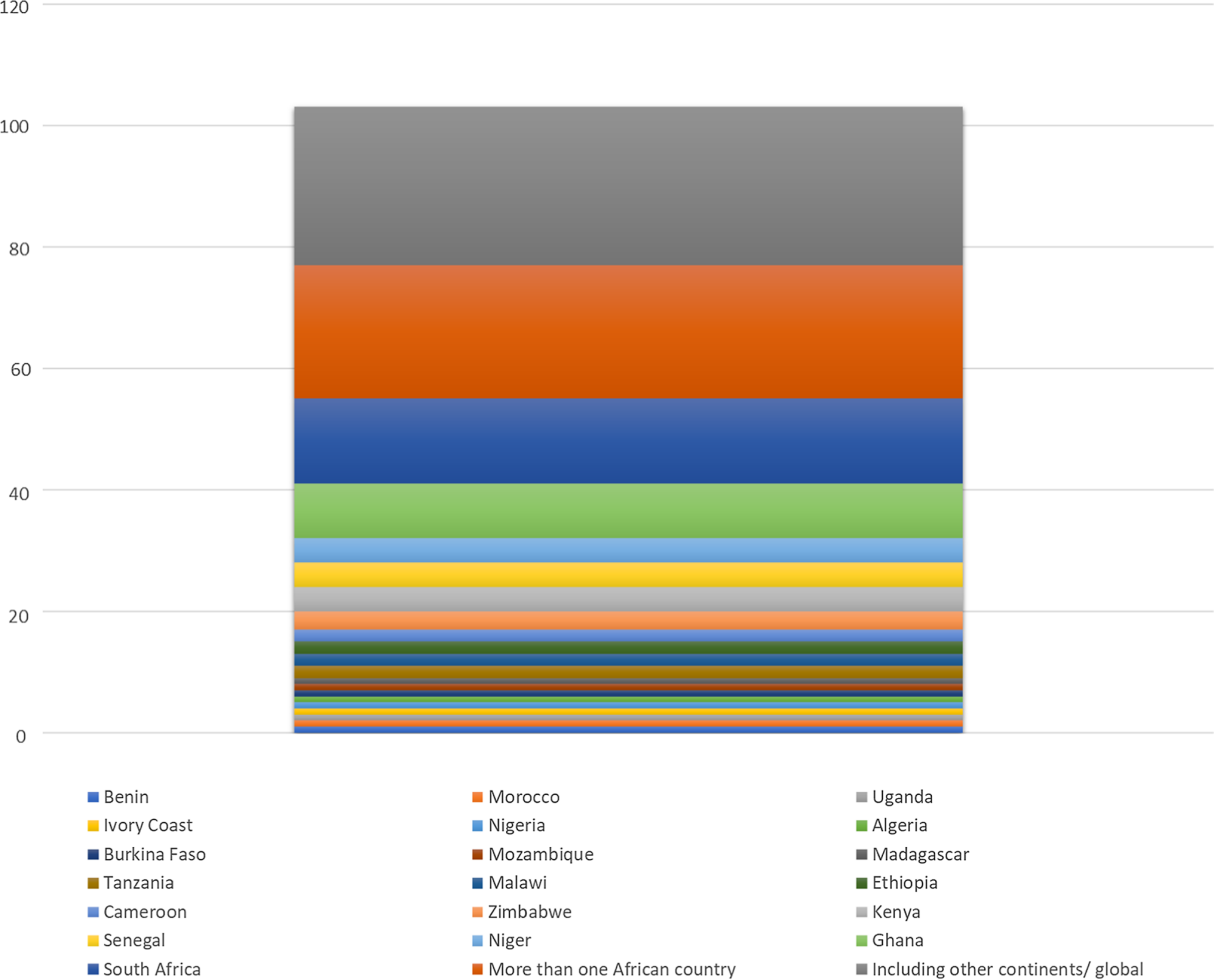
The spatial distribution of studies on this topic, as drawn from a sample of 103 papers published between 2010 and 2021. Source: Author

This study draws from a broad spatial distribution of articles (Fig. 2), from which (n = 77) 74% of the papers were exclusively about Africa, and the remaining 26% included Africa or at least one of its countries in its assessment. Of the 74% of studies that researched Africa exclusively, twenty-three (n = 23) studies explored individual West African countries, n = 13 East African countries, n = 2 North African countries, n = 17 South African countries. N = 22 researched the entire continent, more than one country in the same or different regions of the continent.

**Fig. 3:**
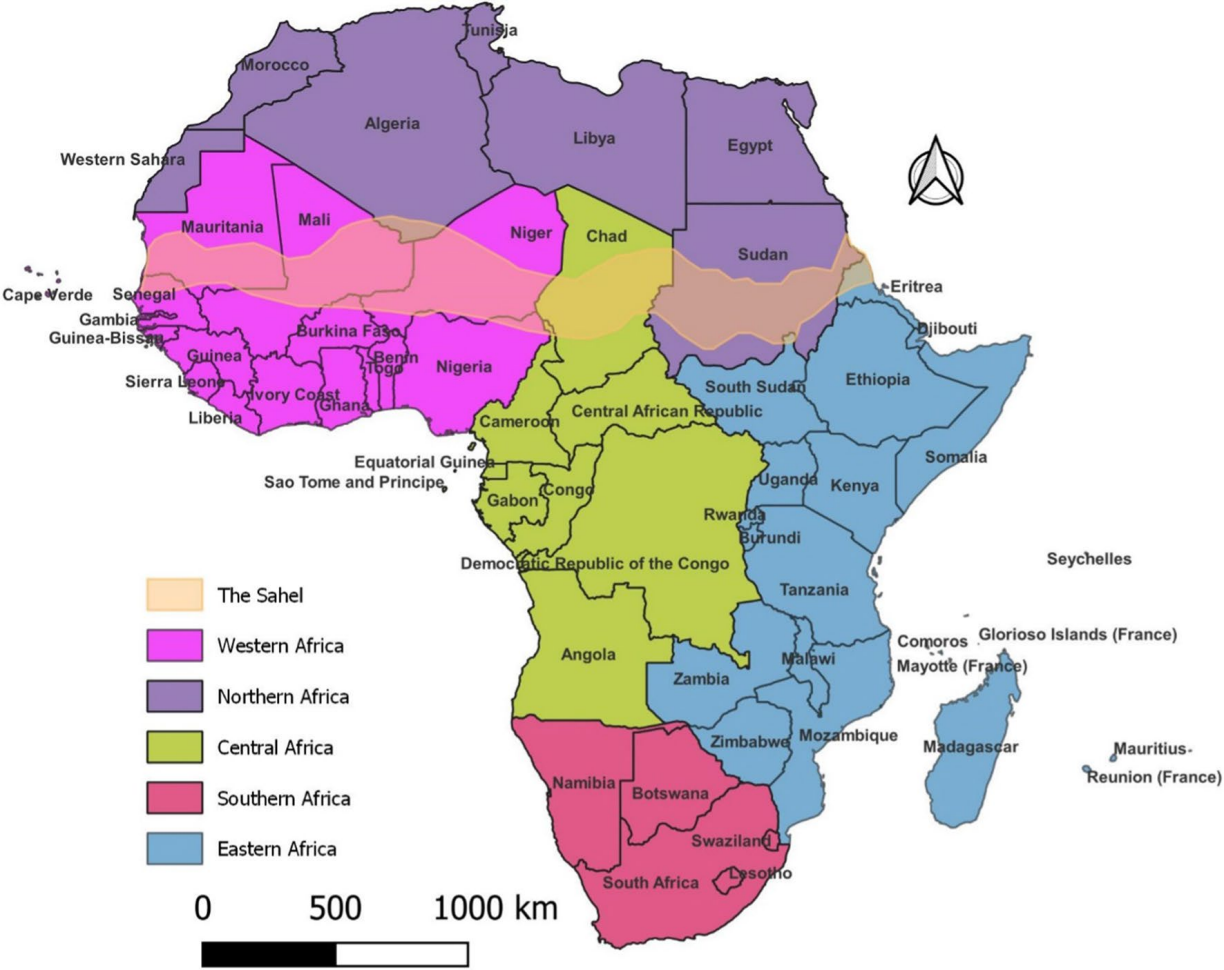
The Sahel and the African countries by region according to the United Nations geoscheme for Africa. Data obtained from https://unstats.un.org/unsd/methodology/m49/#geo-regions, https://www.efrainmaps.es/english-version/free-downloads/world/. Created by: Author

The distribution of African countries by regions, as well as the Sahel, is depicted in Fig. 3.

#### Trend of publication

**Fig. 4:**
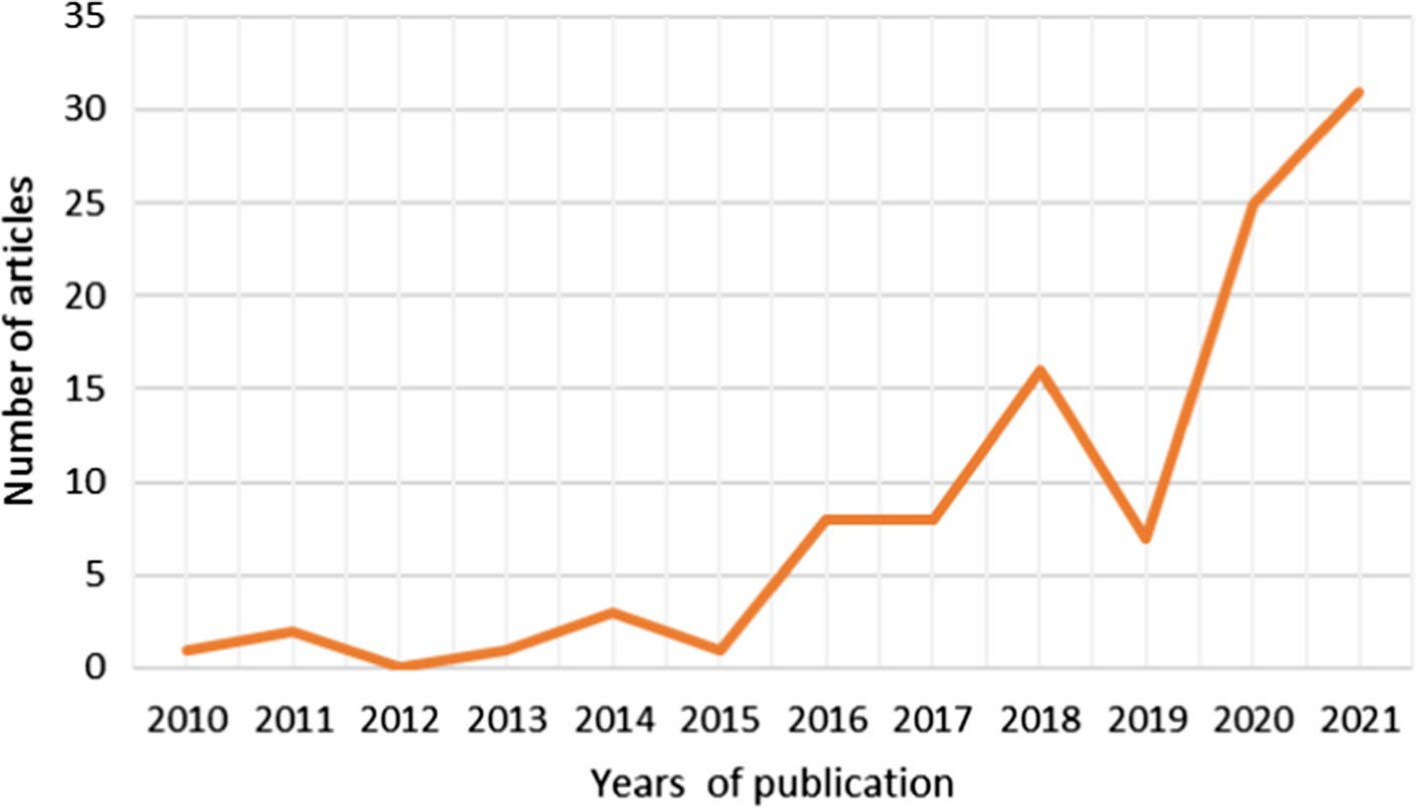
The trend of publication of studies on this topic, as obtained from a sample of 103 papers published between 2010 and 2021, Source: Author

The publication trend of papers relating to flooding, COVID-19, and flood management in a multi-hazard context, in or relating to Africa, shows a sharp increase between 2020 and 2021. The majority of the studies (n = 31) were published in 2021, representing 30% of the total publication years reviewed. followed by 2020 (n = 25), representing 24%, and 2018 (n = 16), representing 15% of the total publication years included in this study. Figure 4 shows the trend of publication of the reviewed papers.

#### Methods employed

**Fig. 5:**
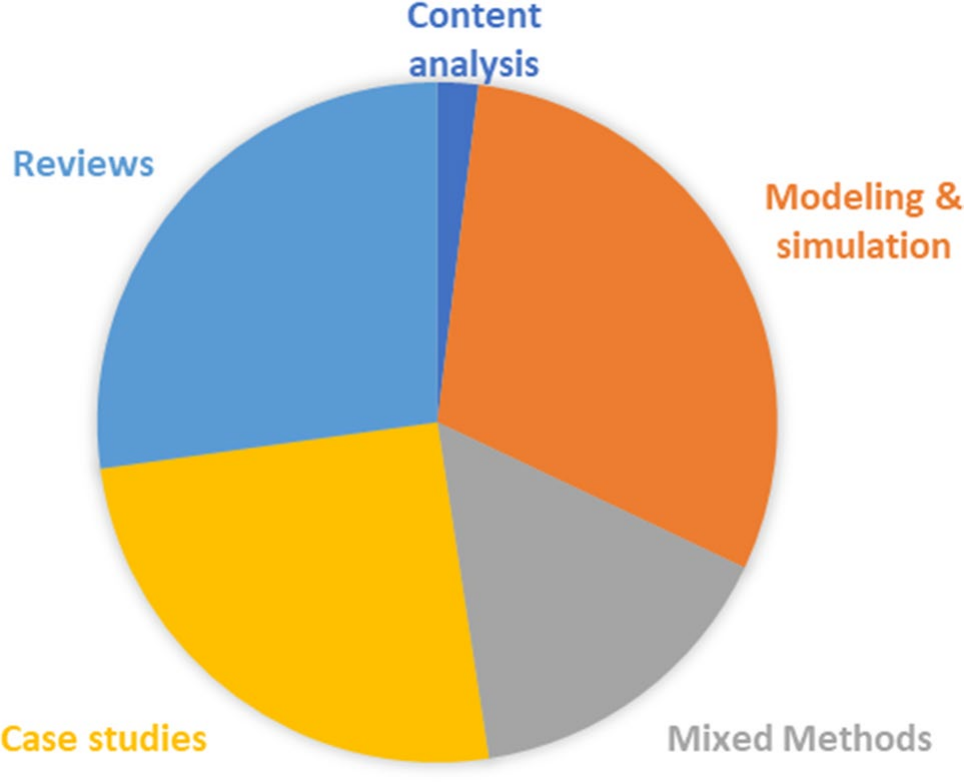
The methodologies applied by studies on this topic as drawn from a sample of 103 papers, published between 2010 and 2021. Source: Author

The methods the studies used were accessed. The result showed that many studies (n = 57) used qualitative methods, representing 55% of the total studies. Thirty-one studies used quantitative methods, accounting for 30% of the sample. The remaining 15% of the studies utilized mixed methods research. The qualitative research methods employed are case studies (26), content analysis (n = 2), and descriptive/conceptual reviews (n = 29). 31 studies used modelling/ simulation quantitative methods. Figure 5 shows the share of methodologies applied by the studies I reviewed.

### Hazards

**Fig. 6:**
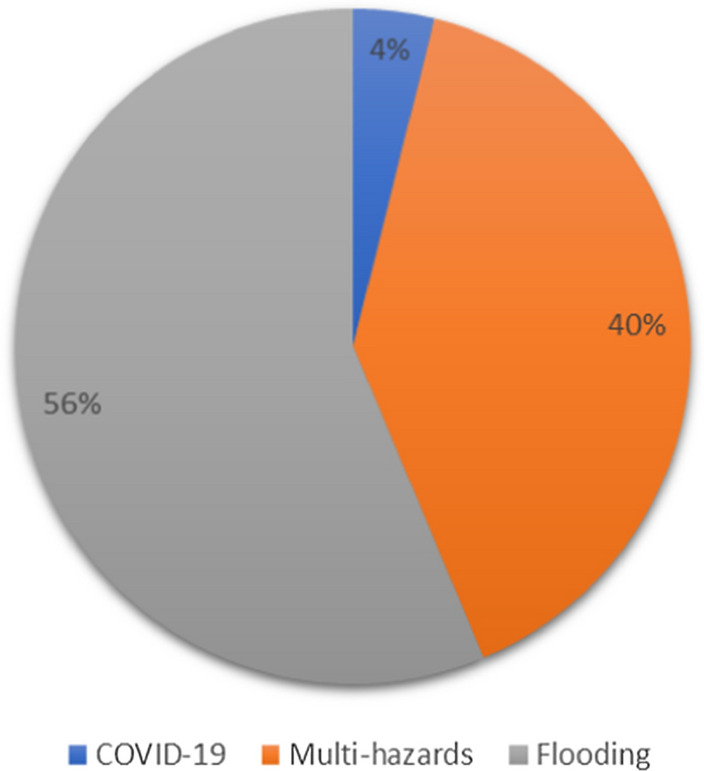
Hazards investigated in this topic as drawn from a sample of 103 papers, published between 2010 and 2021. Source: Author

To get insights into multiple hazards circumstances, the author accessed whether the hazards were studied singularly or in combination (Fig. 6). Studies that investigated one hazard (n = 68) accounted for 65% of the total, shared between flooding (64%) and COVID-19 (4%). The rest of the papers (35%) researched the combination of two or more hazards. Of the 36 articles investigating more than one hazard, 15 were exclusively about Africa. Of these 15 papers, eight investigated two hazards only, while the rest investigated more than two. Of the literature that studied multiple hazards in Africa, floods and droughts were mostly researched together.

Results from the literature review show that droughts and floods are the adversities the east African region is frequently battling with [14, 35, 40]. In addition to flooding and drought conditions impacting the East African region, the locust outbreak is another atrocity [16], having life-threatening impacts [33]. With all these threats in the region, there are still uncertainties in relevant decision-making [6].

Rains do not occur as frequently in arid Northern Africa as they do in other parts of the world, but they have a significant impact when they do. However, floods can also benefit the region by recharging desiccated soils and underground water storage [15, 36]. Aside from material destruction, several aspects of repercussions from flood risk in -Western Africa include health impacts and economic losses [44]. The South-Eastern part of Africa has previously been hit by severe cyclones, exacerbating the COVID-19 disease and flooding damage [5, 10]. Poor communities are defenceless due to the exposure of their livelihoods to flood impacts, particularly in rural locations with limited access to services and infrastructure [19]. However, exposure to flood damage has little difference between rural and urban areas in Sub-Saharan Africa. Rapid population growth in urban areas puts buildings in flood-prone areas, often without suitable infrastructure [30].

### What are the recommendations for managing floods during the COVID-19 pandemic?

**Fig. 7:**
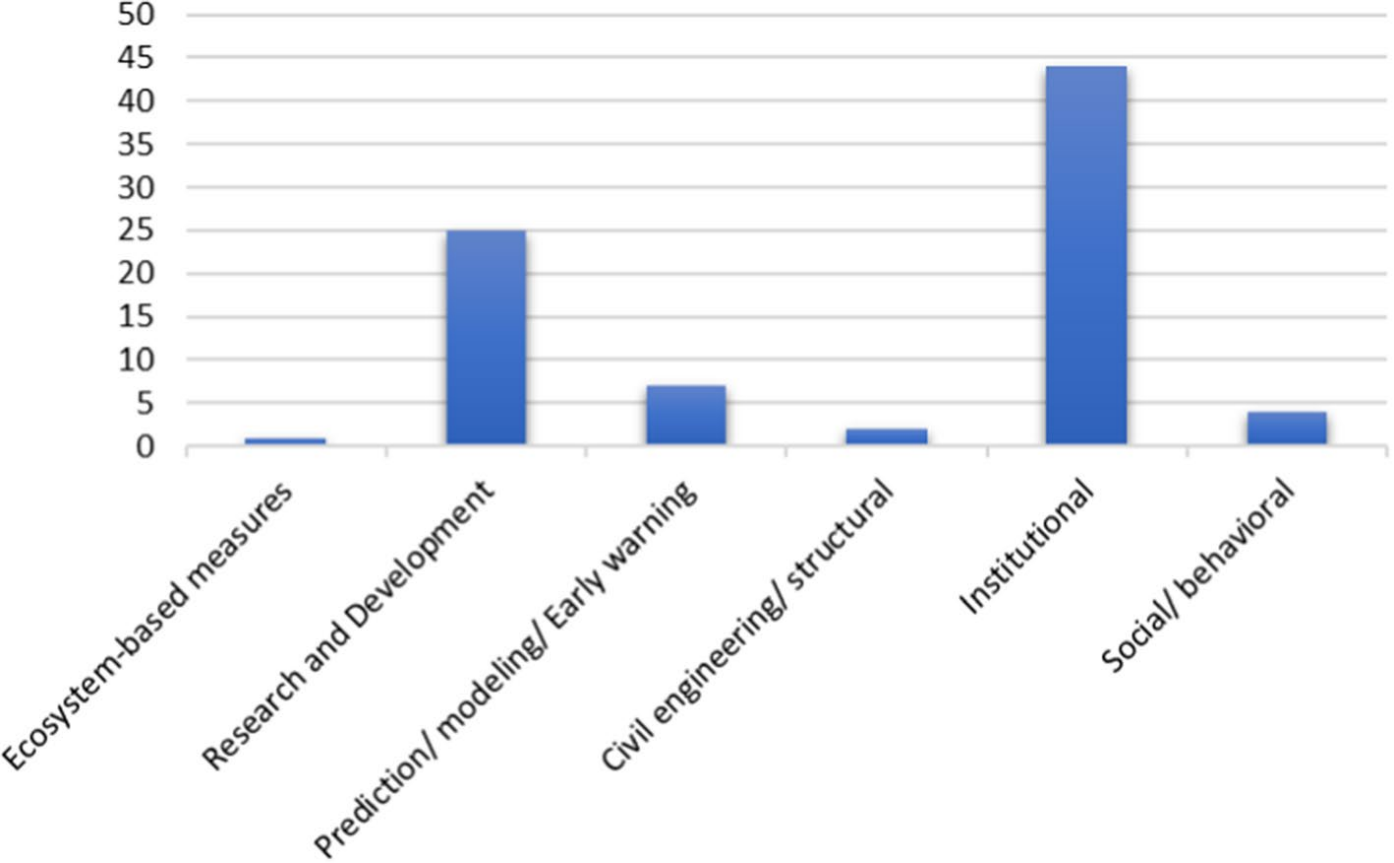
Authors' recommendations in managing floods as obtained from a sample of 103 papers, published between 2010 and 2021. Source: Author. Note that recommendations from one paper could fall into two or more categories

Recommendations of relevant studies reviewed, published on and after the WHO's declaration of COVID-19 as a global pandemic (on March 11, 2020), were analyzed to determine their suggestions for managing floods (Fig. 7). Of the total (n = 104) papers, the author assessed the recommendations from 39 articles fitting into the requirements above, categorising them into Ecosystem-based measures, Research and Development, Prediction/modelling/ Early warning, Civil engineering/ structural, Institutional, Social/behavioural.

#### Institutional

Recommendations about national governments and international institutions taking the lead in effectively managing floods cover 55% of the total (n = 83) recommendations.

In this category, there is a consistency in suggestions that institutions should capacitate local communities to take the lead in responding to and managing local hazards and disasters [3, 45]. Authors also agree that disaster risk management institutional policies should be long-term [3, 11, 34]. Researchers further suggest that inequality should be reduced to minimize impacts and recurring losses during floods and other hazards [18, 29].

Almoradie, in his 2020 publication, intimated that government should strengthen institutional mandates and functions [2], and those mandates and functions should consider diverse inputs and the potential outcome on the entire society amid the ongoing COVID-19 pandemic and frequent flood events [3]. Likewise, in addressing the current multi-hazards, and in preparing for future circumstances in Africa, governments should shift their focus from vulnerability to building resilience [13, 39].

#### Research and development

Recommendations that research and development should be the foundation for managing floods in a multi-hazard context account for 30% of the total recommendations.

Authors suggest that collaboration among intellectual bodies, governments, and project implementers should be strengthened to reduce disaster damage in the African continent and elsewhere [4, 12, 27]. Agreement in recommending that practical knowledge sharing between research and industry can improve flood management innovations in the African continent was also seen in the literature [2, 4].

Pelling advised that climate adaptation studies that reflect the socio-economic and cultural contexts on equal terms with the dynamics of climate systems and hazards in framing risks toward climate-resilient development should be prioritized [27].

To prepare for and mitigate the potential impacts of a multi-hazard problem, in-depth research is suggested to be done in understanding systemic risks, dynamic vulnerability, and the root causes of risks [27, 44].

Ranger [31] added that loss and damage scenarios should be devised and integrated into fiscal and financial risk management frameworks. This move could improve economic resilience and help mitigate future crises [31].

Future research and development projects on multiple hazard circumstances in Africa should be on a common platform that incorporates and encourages the ideas of all stakeholders at all stages [13]. This step could help with bridging gaps and avoiding inefficiencies and mistakes.

#### Prediction/modeling/early warning

Researchers suggest that hazard managers should give attention to the modelling and prediction of hazards, and in strengthening early warning systems account for 9%. They agree that the early detection of catastrophic events will reduce the potential losses and damages [1, 3, 8, 13, 45].

Walton advised that anticipatory plans and actions ahead of climate-related catastrophes should be a primary factor in adapting to climate change and in humanitarian response [45]. Early warning systems should be strengthened to accurately foresee many hazards at one time [3, 28].

A multi-hazard system approach based on simulation, optimization, and multi-objective assessments can help plan for future combined hazard circumstances on the African continent [39].

#### Social/behavioural

Researchers put forward some social/behavioural suggestions for managing floods during the COVID-19 pandemic. These suggestions account for 4% of the total. To ensure effective hazard management, measures should go side-by-side with society's customs and needs, and people should be flexible to change their behaviours to reduce vulnerability and exposure to hazards [3, 7, 38, 39].

Hazard management, according to Borowski, should try to affect risk perception in informed and acceptable ways today and in the future [7].

Measures to manage floods could fail without paying attention to and respecting social and behavioural diversity. In any hazard circumstance, it is crucial that responders in Africa design response strategies that are culturally sensitive and can adapt to uncertainties in human behaviours [7, 39].

#### Civil engineering/ structural

Two per cent (2%) of the recommendations emphasized the importance of constructing and strengthening structures that can withstand flood damage during this pandemic.

Mzava proposed in his study that hydrologic engineering in Dar es Salaam should be engineered to be adapted to both current and future hazards [25]. Buildings that can adapt to climate hazards are considered one of the most effective means to reduce the impacts of flood hazards during the COVID-19 pandemic and even in future circumstances in Africa [13, 25].

#### Ecosystem-based measures

Ecosystem-based measures suggested are the least, with 1%. According to Samu and Akıntuğ [35], investing in ecosystem-based initiatives like mangrove planting and conservation can help reduce cyclone and flood damage while increasing the potential for a robust and green recovery [13, 44].

Climate-related catastrophes such as flooding can be mitigated by investing in ecologically friendly agricultural practices, as well as the conservation and restoration of forests, wetlands, coral, and oyster reefs [35, 44].

### Flood hazard management during the COVID-19 pandemic in Africa

The author took account of how the literature reported flood management activities during the pandemic in the continent.

The various flood management activities were categorized as structural and non-structural and allocated under the African implementation regions. Structural flood management activities (n = 11) include digging drainages and filling sandbags. Non-structural activities (n = 25) include regulations, training, information, and communication to reduce flood impacts. The majority (83%) of the flood management operations reviewed were carried out in East African countries. South and Western Africa each have 8.5 per cent of the total flood management activities reviewed.

Most of the flood mitigation activities in South and Eastern Africa that occurred during the COVID-19 pandemic went hand in hand with combatting locust outbreaks and their impacts in the regions. The Red Cross and Red Crescent (RCRC) supported governments in providing water purification supplies in Ethiopia, Zambia, and Mozambique following flood damage. The state offered insecticide-treated bed nets, restrooms, and community water facilities in Zambia with the RCRC support [42]. The government also provided cash and shelter kits for flood victims in Uganda with the RCRC backing [42]. Disaster damage is done by restricting settlements in flood-prone areas and investing in dikes in South Africa [35]. The government was said to compensate displaced people with land and cash payments in South Africa [24].

According to Kassegn, the government of Ethiopia has initiated the renovation and construction of canals to divert floods [16]. Also, flood early warning is being provided to the flood-exposed population. Additionally, flood management is integrated as part of the county’s water resource policies. And the government is believed to be working on a master plan for all of the country's river basins [16].

Dikes were built using sandbags to strengthen critical infrastructure in Mali and Niger following the devasting impacts of floods in 2020 [42]. In Ghana, flood incidents most often result in electrical failures. As a result, preventive maintenance is usually done to ensure performance and reliability [2, 26]. The government of Ghana provided free water and subsidized electricity to the public in 2020. This intervention was to mitigate COVID-19's impact but was reported to have also lessened the consequences of floods [2, 23].

## Conclusion

This research has put forward critical knowledge for understanding flood management in a multi-hazard context in Africa during the COVID-19 pandemic.

Amid hazards other than flooding, as covered in this study. I discovered that the authors have a firm agreement in recommending the non-structural enabling environments for managing combined hazards in the African continent.

There is very little attention paid to recommending the use of ecosystem-based measures in flood management, and they are not used in managing flood events. Furthermore, researchers studying floods paid more attention to the impacts and how to prevent reoccurrences. However, whether or not the floods were successfully managed was left untreated by the majority of the researchers.

Future research should study how different countries in Africa are preparing to manage multiple future hazards, including the comparative assessment of the strengths and weaknesses of individual countries’ planning and preparation. It will be necessary for each country on the African continent to have a free or subsidized open-access journal with a hazard and disaster management scope. This approach will encourage more researchers into hazards and disaster risks management, providing an enabling platform to unearth hidden challenges and opportunities in managing floods and other hazards on the continent.
